# Detection of minimal hepatic encephalopathy in patients with cirrhosis based on the Stroop‐CN model (NCRCID‐CHESS 2106): a prospective multicenter study

**DOI:** 10.1002/mco2.627

**Published:** 2024-07-15

**Authors:** Xiaoyan Li, Shanghao Liu, Ying Guo, Hongmei Zu, Huiling Xiang, Shaoqi Yang, Xiaoning Zhang, Fanping Meng, Yangzhen Bianba, Jie Li, Fei Liu, Chuang Lei, Jiaojian Lv, Qiao‐hua Yang, Wei Fu, Wei Ye, Jiafang Chen, Yanjing Gao, Caiyun Wu, Ningning Wang, Qi Zheng, Fang Wang, Jiali Yu, Jing Wang, Xiaoting Yang, Xiangmei Wang, Yayuan Liu, Xuelan Zhao, Chenxi Wu, Wei Gou, Jasmohan S. Bajaj, Fu‐Sheng Wang, Junliang Fu, Xiaolong Qi

**Affiliations:** ^1^ Medical School of Chinese PLA Beijing China; ^2^ Senior Department of Infectious Diseases The Fifth Medical Center of Chinese PLA General Hospital, National Clinical Research Center for Infectious Diseases Beijing China; ^3^ Center of Portal Hypertension Department of Radiology Zhongda Hospital, Medical School, Southeast University, Nurturing Center of Jiangsu Province for State Laboratory of AI Imaging & Interventional Radiology (Southeast University) Nanjing China; ^4^ Basic Medicine Research and Innovation Center of Ministry of Education Zhongda Hospital Southeast University Nanjing China; ^5^ State Key Laboratory of Digital Medical Engineering Nanjing China; ^6^ Department of Hepatology The Third People's Hospital of Taiyuan Taiyuan China; ^7^ Department of Gastroenterology The Fourth People's Hospital of Qinghai Province Xining China; ^8^ Department of Gastroenterology and Hepatology Tianjin Third Central Hospital, Tianjin Key Laboratory of Extracorporeal Life Support for Critical Diseases, Institute of Hepatobiliary Disease Tianjin China; ^9^ Department of Gastroenterology The General Hospital of Ningxia Medical University Yinchuan China; ^10^ Department of Hepatology The Third People's Hospital of Tibet Autonomous Region Lhasa China; ^11^ Department of Infectious Diseases Nanjing Drum Tower Hospital, The Affiliated Hospital of Medical School Nanjing University Nanjing China; ^12^ Department of Infectious Diseases Hunan Key Laboratory of Viral Hepatitis Xiangya Hospital Central South University Changsha China; ^13^ Department of Infectious Diseases The First People's Hospital of Changde City Changde China; ^14^ Department of Infectious Disease Lishui City People's Hospital Lishui China; ^15^ Hepatology Department of Infectious Diseases Center The First People's Hospital of Huaihua Huaihua China; ^16^ Department of Hepatology Shenyang 739 Hospital Shenyang China; ^17^ Liver Cirrhosis Treatment Center Nanjing Hospital Affiliated to Nanjing University of Traditional Chinese Medicine Nanjing China; ^18^ Department of Gastroenterology Datong City Fourth People's Hospital Datong China; ^19^ Department of Gastroenterology Qilu Hospital of Shandong University Jinan China; ^20^ Department of Hepatology Third People′s Hospital of Linfen City Linfen China; ^21^ Department of Gastroenterology The First Hospital of China Medical University Shenyang China; ^22^ Department of Hepatology Hepatology Research Institute The First Affiliated Hospital Fujian Medical University Fuzhou China; ^23^ Department of Hepatology Shenzhen Third People's Hospital, National Clinical Research Center for Infectious Disease, The Second Affiliated Hospital, School of Medicine, Southern University of Science and Technology Shenzhen China; ^24^ Department of Gastroenterology The First Affiliated Hospital of Dalian Medical University Dalian China; ^25^ Department of Gastroenterology The Second Affiliated Hospital of Baotou Medical College Baotou China; ^26^ Department of Gastroenterology Wuzhong People's Hospital Wuzhong China; ^27^ Department of Severe Hepatology Mengchao Hepatobiliary Hospital of Fujian Medical University Fuzhou China; ^28^ Department of Gastroenterology Central hospital of Dandong Dandong China; ^29^ Department of Gastroenterology Liver Diseases Chongqing Public Health Medical Center Chongqing China; ^30^ Liver Disease Diagnosis and Treatment Center Yiyang Fourth People's Hospital Yiyang China; ^31^ Qingdao Sixth People's Hospital Qingdao China; ^32^ Division of Gastroenterology, Hepatology and Nutrition Virginia Commonwealth University and Central Virginia Veterans Healthcare System Richmond Virginia USA

**Keywords:** cirrhosis, minimal hepatic encephalopathy, quality of life, Stroop test

## Abstract

Minimal hepatic encephalopathy (MHE) has a substantial impact on the clinical outcomes and quality of life (QOL) of patients with cirrhosis. However, timely diagnosis and intervention are challenging due to sophisticated diagnostic methods. In this study, 673 healthy controls and 905 patients with cirrhosis were screened, and 660 healthy controls and 757 patients with cirrhosis, divided into the test (292 patients) and validation (465 patients) cohort, were analyzed after screening. A diagnostic model of the Stroop test (Stroop‐CN) was constructed by multivariate linear regression based on the results of healthy controls. The prevalence of MHE and the comparison results with psychometric hepatic encephalopathy score through the Stroop‐CN model were stable in the test and validation cohorts. Moreover, the prevalence of MHE remained significantly higher in patients with worse disease conditions marked as high Child–Pugh grades and the Model for End‐stage Liver Disease and Sodium (MELD‐Na) scores in the test and validation cohort. The EuroQol 5‐D questionnaire revealed that patients with MHE had a worse QOL than those without MHE both in the test and validation cohort. In conclusion, an easy and practical Stroop‐CN model for MHE diagnosis based on the EncephalApp is established. It is found that a considerable number of Chinese patients with cirrhosis experience MHE, which significantly impacts their QOL.

## Graphical abstract

The Stroop‐CN model was developed based on health control results and was used to identify MHE patients among cirrhotic patients. This subset of MHE patients had significantly lower quality of life than non‐MHE patients.

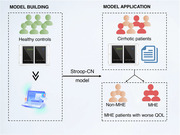

## 1 INTRODUCTION

Hepatic encephalopathy (HE) is a vital comorbidity of cirrhosis present in 50%−80% of patients during their natural history and brings high mortality.^1^, ^2^ Minimal hepatic encephalopathy (MHE) is characterized by neuropsychological disorders and occurs in 35%–55% patients with cirrhosis, but does not typically involve behavioral abnormalities.^1^, ^3^ Previous studies have shown that MHE patients have a high risk for falls, car crashes, overt HE, and even death, affecting their quality of life (QOL).^4^, ^5^, ^6^, ^7^ The neuropsychological impairment in MHE patients mainly manifests in psychomotor and sensitivity, processing speed, spatial orientation, and other neuropsychological functions.^8^ Recent global guidelines recommend using the psychometric hepatic encephalopathy score (PHES) to diagnose and forecast outcomes of MHE in cirrhotic patients, as it assesses the mental functions mentioned above.^1^, ^2^, ^9^ However, the PHES consists of five paper–pencil subtests that require 20–30 min under professional instruction, which is time consuming for patients and physicians and limits the clinical application. Therefore, many patients with MHE still do not receive appropriate diagnosis and treatment. More efficient and user‐friendly methods are urgently needed.

The Stroop test, which can reflect the processing speed, cognitive flexibility, selective attention, and executive function of the subject by quickly selecting the correct color of the symbol or word, has been applied to diagnose MHE.^10^, ^11^, ^12^ Compared with PHES, the Stroop test can be finished in 10 min with a smart device, making it more convenient, easy to operate, and patient‐friendly. Previous studies have demonstrated the high sensitivity and reliability of the Stroop test in diagnosing MHE and predicting the onset of overt HE.^10^, ^11^, ^12^, ^13^, ^14^, ^15^, ^16^, ^17^, ^18^ However, studies found that the results of the Stroop test correlate with the age, sex, and education level of the subjects in the same way as PHES, suggesting that the inherent features of the population cognition may affect the neuropsychological tests’ performance.^1^, ^12^, ^14^, ^15^, ^17^, ^18^, ^19^ In addition, regional literacy level and economic status can also vary the Stroop test results. Therefore, it is more reasonable to apply the Stroop test to the diagnosis of MHE according to the diagnostic criteria established based on the local healthy population.

As a nation facing a significant prevalence of liver diseases, China has an estimated over one‐fifth of its population affected by various forms of liver disease, with 7 million patients suffering from cirrhosis, resulting in 160,000 all‐cause deaths by cirrhosis per year.^20^, ^21^ With the advancement in socioeconomic and the treatment of diseases, the etiology spectrum of liver disease has also changed dramatically. Therefore, it is necessary to use an easy and practical diagnostic method to determine the current prevalence of MHE. Previous studies on the prevalence of MHE in China have mainly used PHES as the diagnostic tool, and there is a lack of investigation based on the Stroop diagnosis model through healthy controls, and the impact of MHE on patients’ QOL needs to be further clarified.^13^, ^18^, ^22^, ^23^ This study aimed to create a diagnostic model named Stroop‐CN for MHE, utilizing Stroop results from a large cohort of healthy individuals, and to evaluate the prevalence and effects of MHE in patients with cirrhosis.

## 2 RESULTS

### 2.1 Demographic characteristics of all participants

**FIGURE 1:**
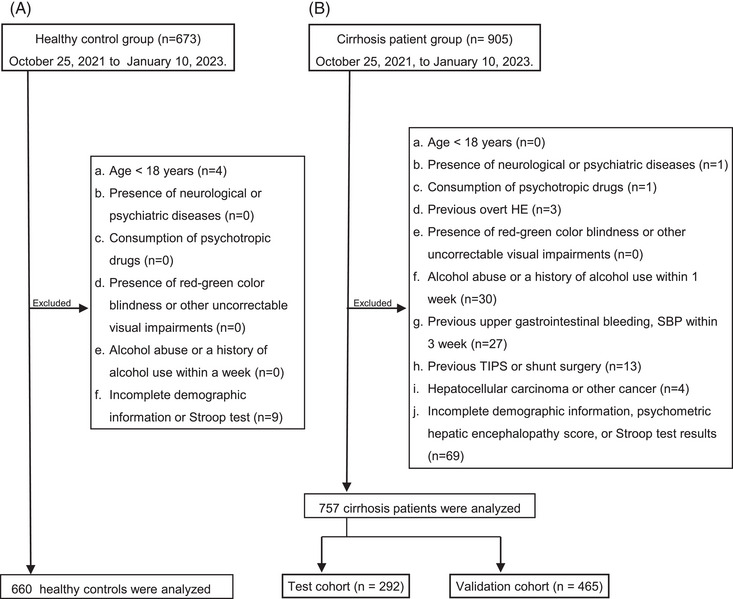
Enrollment flow chart of the study. HE, hepatic encephalopathy; SBP, spontaneous bacterial peritonitis; TIPS, transjugular intrahepatic portosystemic stent shunt.

A total of 673 healthy controls and 905 patients with cirrhosis were screened from 27 hospitals between October 25, 2022, and January 10, 2023. According to the study criteria, 660 healthy controls and 757 patients were included in further analysis, and patients were divided into the test cohort (*n* = 292) and validation cohort (*n* = 465). The patient selection flowchart is illustrated in Figure 1. The age and duration of education were significantly different between healthy controls and patients with cirrhosis (both *p* < 0.001) (Table 1). There are notable variations in gender and duration of education across all age groups for both healthy controls and patients with cirrhosis, with all differences being statistically significant (all *p* < 0.05). Chronic hepatitis B (73.2%) was the main cause of cirrhosis in our patients, followed by autoimmune liver diseases (7.0%). The Model for End‐stage Liver Disease and Sodium (MELD‐Na) scores of the patients with available information were 10.0 (8.0, 14.0) (Table 1).

**TABLE 1:** Baseline characteristic of healthy controls and patients with cirrhosis.

	Healthy controls (*n* = 660)	Liver cirrhosis (*n* = 757)	*p* value
Age (year)	35.0 (30.0, 46.5)	52.0 (45.0, 58.0)	<0.001
Sex (male/female)	189/471^a^	489/268^a^	<0.001
18−29 years (male/female)	32/120	10/7	
30−39 years (male/female)	63/208	73/12	
40−49 years (male/female)	44/58	143/55	
50−59 years (male/female)	32/59	193/125	
≥60 years (male/female)	18/26	70/69	
Education (year)	16.0 (12.0,17.0)^a^	9.0 (6.0,12.0)^a^	<0.001
18−29 years	16.0 (16.0, 16.0)	14.0 (7.0,15.0)	
30−39 years	16.0 (15.0, 18.0)	11.0 (9.0,14.0)	
40−49 years	15.5 (10.0, 16.0)	9.0 (6.0,12.0)	
50−59 years	12.0 (9.0, 16.0)	8.0 (5.0,12.0)	
≥60 years	11.0 (9.0, 12.0)	9.0 (6.0,12.0)	
Etiology of cirrhosis			
Chronic hepatitis B	–	554 (73.2%)	–
Autoimmune liver diseases	–	53 (7.0%)	–
Chronic hepatitis C	–	51 (6.7%)	–
Alcoholic liver diseases	–	29 (3.8%)	–
Others^b^	–	70 (9.3%)	–
Child–Pugh A/B/C/no data	–	420/215/82/40	–
MELD‐Na score^c^	–	10.00 (8.00, 14.00)	–
MELD‐Na <10/10–20/>20/no data	–	356/113/24/264	–

^a^ There were significant differences among the following different age groups (*p* < 0.001).

^b^ Other etiologies included drug‐induced, metabolic associated, toxic‐induced, and cryptogenic.

^c^ Data were obtained from 493 patients.d.MELD‐Na, the Model for End‐stage Liver Disease and Sodium score

### 2.2 Results of healthy controls and Stroop‐CN model

The results of the Stroop test in the healthy control group are presented in Table 2. Spearman analysis showed that the off time, on time, and off + on time results were significantly correlated with age and duration of education, and the Mann–Whitney *U* test showed that the above results had significant differences between sexes, with better results in female (all *p* < 0.05) (Table 3). Results also showed a notable relationship between number of runs and duration of education. Details are shown in Table 3.

**TABLE 2:** The results of Stroop test in healthy controls and patients with cirrhosis.

	Healthy controls	Patients with cirrhosis	*p*
Off time (s)	68.839 (60.587, 80.736)	94.225 (80.000, 117.988)	<0.001
On time (s)	79.195 (68.731, 94.098)	108.656 (90.407,131.084)	<0.001
Off + on time (s)	150.069 (130.592, 174.666)	204.992 (174.033, 245.998)	<0.001
Off runs (times)	5 (5, 6)	5 (5, 6)	0.286
On runs (times)	6 (5, 7)	6 (5, 7)	0.274
On—off time (s)	9.581 (4.225, 16.530)	10.277 (2.074, 22.069)	0.355

**TABLE 3:** Correlation of the Stroop results with sex, age, and duration of education in the healthy control group.

	Sex		Age (years)		Education (years)	
	*z*	*p*	*r*	*P*	*r*	*p*
Off time (s)	−2.896	0.004	0.439	<0.001	−0.305	<0.001
On time (s)	−2.432	0.015	0.380	<0.001	−0.215	<0.001
Off + on time (s)	−2.579	0.010	0.426	<0.001	−0.271	<0.001
Off runs (times)	−1.812	0.070	‐0.062	0.114	−0.030	0.436
On runs (times)	−0.607	0.544	0.017	0.671	−0.138	<0.001
On—off time (s)	−1.243	0.214	0.000	0.998	0.046	0.236

*Note*: The differences of results between the sexes were tested by Mann–Whitney *U* test. The correlations between results and age or duration of education were tested by Spearman analysis.

Multivariate linear regression was performed on the age, sex, and duration of education to construct the expected value equations for the off + on time according to the results of healthy controls. The expected value model was off + on time (Stroop‐CN model) = 144.332 + 1.921 × age − 3.665 × education. According to the age and duration of education of patients, the expected off + on time can be calculated through the model, and patients whose measured results were worse than the expected value were considered to have MHE.

### 2.3 Prevalence of MHE in the test and validation cohort

The Stroop test results for patients with cirrhosis are displayed in Table 2. There were significant differences in the off time, on time, and off + on time results between healthy controls and patients with cirrhosis (all *p* < 0.001) (Table 2). To further validate the stability of the Stroop‐CN model, the patients were divided into the test and validation cohorts. The comparison results of baseline data between the two groups are displayed in Table 4. There were no differences between the results of the PHES and Stroop tests in the test and validation groups, as shown in Table S1. According to the Stroop‐CN model, 44.9% (131/292) and 49.0% (228/465) of cirrhotic patients had worse results in terms of off + on time than expected, resulting in a diagnosis of MHE in the test and validation cohort, respectively. Results indicated that there were no significant differences between the two groups regarding MHE prevalence, demographic characteristics, and etiologies of cirrhosis (all *p* > 0.05) (Table 4).

**TABLE 4:** Characteristics of test and validation cohort in patients with cirrhosis.

	Test (*n* = 292)	Validation (*n* = 465)	*p* value
Age (year)	52.0 (45.0, 58.0)	52.0 (45.0, 58.0)	0.749
Gender (male/female)	185/107^a^	304/161^a^	0.572
18−29 years (male/female)	5/3	5/4	
30−39 years (male/female)	32/5	41/7	
40−49 years (male/female)	47/24	96/31	
50−59 years (male/female)	24/27	46/42	
≥60 years (male/female)			
Education (year)	9.0 (5.0, 12.0)	9.0 (6.0, 12.0)	0.409
18−29 years (male/female)	13.5 (5.0, 15.0)	14.0 (9.0, 16.0)	
30−39 years (male/female)	12.0 (8.0, 15.0)	9.0 (9.0, 12.0)	
40−49 years (male/female)	8.0 (3.5, 12.0)	9.0 (6.0, 12.0)	
50−59 years (male/female)	8.0 (5.0, 11.0)	9.0 (6.0, 12.0)	
≥60 years (male/female)	9.0 (5.5, 12.0)	9.0 (6.0, 12.0)	
Etiology of cirrhosis			0.786
Hepatitis B	208 (71.2%)	346 (74.4%)	
Autoimmune liver diseases	21 (7.2%)	30 (6.5%)	
Hepatitis C	23 (7.9%)	30 (6.5%)	
Alcoholic	13 (4.5%)	16 (3.4%)	
Others^b^	27 (9.2%)	43 (9.2%)	
Child–Pugh A/B/C/no data	161/82/35/14	259/133/47/26	0.741
MELD‐Na score^c^	10.00 (8.00, 14.00)	10.00 (8.00, 14.00)	0.933
MELD‐Na <10/10–20/ >20/no data	130/41/10/111	226/72/14/153	0.874
MHE by Stroop‐CN (*n*, %)	131 (44.9%)	228 (49.0%)	0.263
MHE by PHES (*n*, %)	102 (34.9%)	182 (39.1%)	0.244

Abbreviations: MHE, minimal hepatic encephalopathy; PHES, psychometric hepatic encephalopathy score, MELD‐Na, the Model for End‐stage Liver Disease and Sodium score.

^a^There were significant differences among the following different age groups (*p* < 0.001).

^b^ Other etiologies included drug‐induced, metabolic associated, toxic‐induced, and cryptogenic.

^c^ Data were obtained from 493 patients.

**FIGURE 2:**
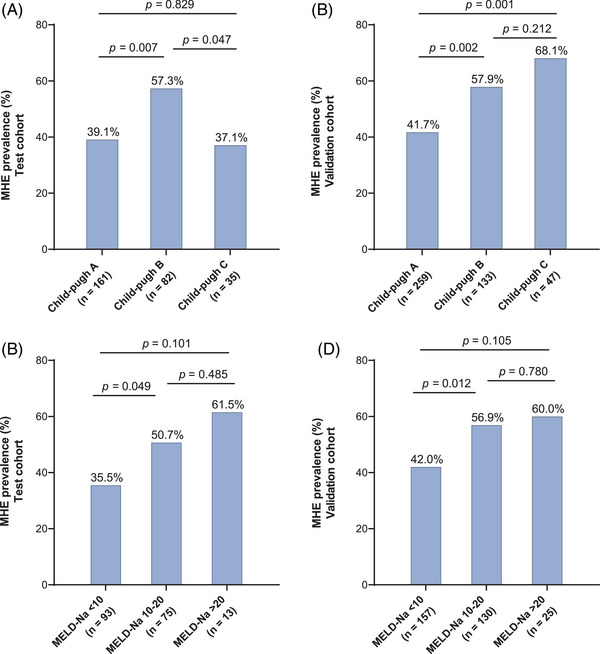
Prevalence of minimal hepatic encephalopathy (MHE) in different groups of patients with cirrhosis. (A and B) Comparison results of MHE prevalence among different Child–Pugh in patients with cirrhosis of test and validation cohort, respectively. (C and D) Comparison results of MHE prevalence among different Model for End‐stage Liver Disease and Sodium (MELD‐Na) score classifications in patients with cirrhosis of test and validation cohort, respectively.

We further investigated the prevalence of MHE in patients with different Child–Pugh grades and MELD‐Na scores. In the test cohort, MHE prevalence was markedly higher in patients with Child–Pugh B (57.3%, 47/82) than in patients with Child–Pugh A (39.1%, 63/161) and C (37.1%, 13/35) (*p* = 0.007 and *p* = 0.047, respectively). However, there was no statistical difference between Child–Pugh A and C groups (*p* = 0.829) (Figure 2A). In the validation cohort, the tendency for MHE prevalence to increase with decreasing liver function was more pronounced. The prevalence of MHE was notably higher in patients classified as Child–Pugh C (68.1%, 32/47) and B (57.9%, 77/133) compared to those classified as Child–Pugh A (41.7%, 122/259) (*p* = 0.001 and *p* = 0.002). However, there was no statistical difference between Child–Pugh C and B groups (*p* = 0.212) (Figure 2B).

In the test cohort, the prevalence of MHE was significantly higher in patients with MELD‐Na score 10−20 (50.7%, 38/75) compared to those with MELD‐Na score < 10 (35.5%, 33/93) (*p* = 0.049). However, there was no statistical difference between patients with MELD‐Na score > 20 (61.5%, 8/13) and those with MELD‐Na score 10−20 (*p* = 0.485), or between patients with MELD‐Na score > 20 and those with MELD‐Na score < 10 (*p* = 0.101) (Figure 2C). In the validation cohort, the prevalence of MHE was significantly higher in patients with MELD‐Na score 10−20 (56.9%, 74/130) compared to those with MELD‐Na score < 10 (42.0%, 66/157) (*p* = 0.012). There was no statistical difference observed between patients with MELD‐Na score > 20 (60.0%, 15/25) and those with MELD‐Na score 10−20 (*p* = 0.780), or between patients with MELD‐Na score > 20 and those with MELD‐Na score < 10 (*p* = 0.105) (Figure 2D).

### 2.4 Patients with MHE have a lower quality of life

**FIGURE 3:**
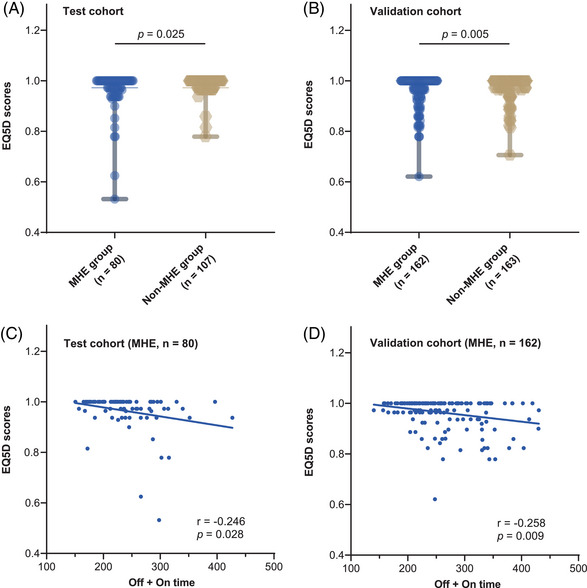
Minimal hepatic encephalopathy (MHE) was associated with decreased quality of life in cirrhosis patients. (A and B) Quality of life was evaluated by EuroQol 5‐D questionnaire (EQ‐5D) in the test and validation cohorts, respectively. (C and D) Correlation between EQ‐5D scores and the off + on time EQ‐5D in the test and validation cohorts, respectively.

Out of the 512 patients with available EuroQol 5‐D (EQ‐5D) results, 187 were part of the test cohort, and 325 were in the validation cohort. In the test cohort, the EQ‐5D score of patients with MHE [1.0000, interquartile range [IQR] (0.9635−1.0000)] was significantly lower than those without [1.0000, IQR (0.9726−1.0000)] (*p* = 0.025) (Figure 3A). Meanwhile, in the validation cohort, the EQ‐5D score of patients with MHE [1.0000, IQR (0.9630−1.0000)] was also significantly lower than those without [1.0000, IQR (0.9726−1.0000)] (*p* = 0.005) (Figure 3B). Pearson analysis showed that the EQ‐5D scores significantly correlated with the off + on time in the MHE group (*r* = −0.246 and −0.258, *p* = 0.028 and 0.009) but not in the non‐MHE group (*r* = −0.072 and −0.028, *p* = 0.463 and 0.722) in the test and validation cohort, respectively (Figure 3C,D).

## 3 DISCUSSION

Previous studies have noted that early intervention in MHE can improve the clinical prognosis of patients or even reverse the MHE state.^24^, ^25^ However, the complex diagnostic approach of MHE is still the main reason that prevents its early diagnosis and treatment. The International Society for Hepatic Encephalopathy and Nitrogen Metabolism recommends that all patients with cirrhosis should be screened for MHE, and even those who do without MHE initially should be re‐tested every 6 months.^1^ However, 38% of AASLD members reported that they had never tested for MHE in their clinical practice, primarily due to the complexity of diagnostic methods.^26^ Compared with other common neuropsychological methods such as PHES, Stroop is simple and easy to use, does not require high cognitive level of the subject, and can do the result page directly after inputting the subject's information. While neurophysiological methods such as electroencephalogram require expensive equipment and specialized personnel for guidance and determination, Stroop requires only an iPad and almost no guidance. This study forms the Stroop‐CN diagnosis model through large‐scale healthy controls, providing a convenient diagnostic method for MHE. Meanwhile, nearly half of patients with cirrhosis were found to suffer from MHE with reduced QOL.

As a convenient test that can be applied to a smart application, Zeng et al. reported that the Stroop test saved 38% of the time compared to the traditional paper–pencil tests.^13^ However, previous studies conducted in different populations have found different optimal diagnostic cutoff values of the Stroop test that range from 186.63 to 274.9 s; meanwhile, even though correlations of results with factors such as age were found, the direct modeling using healthy controls is rarely been established and used.^11^, ^12^, ^13^, ^14^, ^15^, ^17^, ^27^ Bajaj et al. established the Stroop diagnostic model based on the characteristics of the American population, but it cannot be directly applied to patients in other countries.^17^ Zeng's research constructed a model of the Stroop test using the results of 569 healthy Chinese people enrolled from one first‐tier city in China; however, the model has not yet been used to determine MHE in patients with cirrhosis in their subsequent analysis. In addition, their health controls were collected from cities with higher economic levels, which may affect the representativeness of the model.^18^ Our study included healthy controls that covered various demographic conditions from different regions in China to construct the Stroop‐CN model and further practical use to detect prevalence in patients.

**FIGURE 4:**
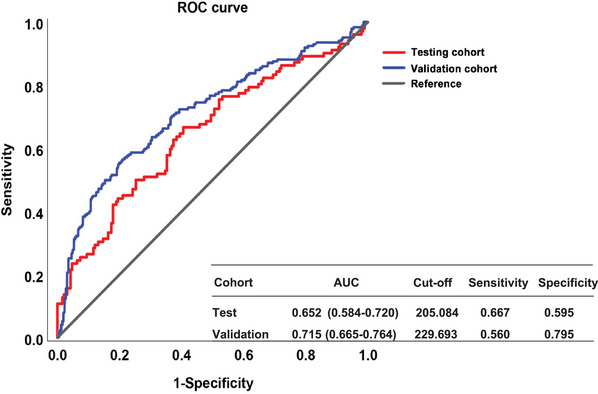
Comparison results of Stroop‐CN with psychometric hepatic encephalopathy score (PHES) in test and validation cohort.

The prevalence of MHE based on the Stroop test differed in the previous studies and ranged from 32.9% to 59.3%.^11^, ^12^, ^13^, ^14^, ^15^ First, for the few studies that determined MHE diagnosis based on the model of expected value, the characteristics of the healthy controls directly affect the model and then further affect the MHE prevalence results. Second, for studies that used the cutoff value derived from other neuropsychological test results, the type of neuropsychological test referenced may influence the cutoff values and the final diagnosis. Our results also confirm that the agreement between the PHES and the Stroop test is not too perfect (see Figure 4). Indeed, there are several aspects of impaired cognitive functioning in patients with MHE, and it is clear that the cognitive deficits detected by different tests do not align, possibly accounting for the varying results obtained from these tests. Still, the consistent prevalence rates and the comparison of the two methods we obtained in the test and validation groups strongly demonstrate the robustness of the Stroop‐CN model (Table 4).

Furthermore, the diverse demographic features of the patients in each study, including variables such as age, education level, etiologies, the severity of cirrhosis, previous medical histories such as overt HE and liver cancer, and the year in which the research was conducted, impact the findings of MHE prevalence determined by the Stroop test. In the current study, we found that the MHE prevalence was associated with the severity of liver function of patients, such as Child–Pugh grades and Meld‐Na scores, even though some of the comparisons got no statistically significant, which is consistent with previous studies.^28^, ^29^, ^30^, ^31^

Simplification of the Stroop test has also been explored, although the Stroop is time saving compared to traditional paper–pencil tests.^32^, ^33^ These studies found that two runs in the off or on state had approximated overall diagnostic values compared with the complete Stroop test, with an Area Under Curve of 0.791 and 0.877, respectively, and can be completed in 1 min. However, both studies included patients with prior overt HE and did not include healthy controls. At the same time, the problem of retaining only two runs of the off or on state is that the psychological function corresponding to the other state cannot be measured. Further validations are still required for the simplified version of the Stroop test.

Our study found QOL impairment in patients with MHE, consistent with previous reports.^16^, ^17^, ^34^, ^35^ Moreover, the off + on time results showed a significant correlation with EQ‐5D scores in the MHE groups but not in the non‐MHE groups, further suggesting that the cognitive impairment reflected by the prolonged off + on time is related to the reduced QOL of patients. In particular, the above results were consistent in both the test and validation cohorts, which reinforces the existence of impaired QOL in MHE patients.

There are some limitations to the study. First, we excluded the patients with previous overt HE and the presence of severe comorbidities, which are a large proportion of the population in practice. Second, some patients in our study lacked Child–Pugh grades, MELD‐Na score, and EQ‐5D information, which may have caused some bias in the subgroup analysis.

In conclusion, this prospective, multicenter study established the Stroop‐CN model for MHE diagnosis based on a large sample of healthy people in China and found that MHE was present in nearly half of the patients with cirrhosis, which further resulted in reduced QOL for patients. Our results provide a reference for exploring a generally applicable diagnostic and monitoring method for MHE.

## 4 MATERIALS AND METHODS

### 4.1 Study population

This prospective, multicenter study was initiated by the National Clinical Research Center for Infectious Diseases (NCRCID) and the Portal Hypertension Alliance in China (CHESS). All participants were enrolled in 27 hospitals in China between October 25, 2021, and January 10, 2023. This study is reported according to the “Strengthening the Reporting of Observational Studies in Epidemiology” checklist.^36^

The following exclusion criteria apply to all participants including both healthy controls and patients: (1) aged ≤ 18 years; (2) presence of neurological or psychiatric diseases such as schizophrenia, bipolar disorder, depression, anxiety, cerebral infarction, and cerebral hemorrhage; (3) consumption of psychotropic drugs such as sedatives, hypnotics, and antidepressants; (4) presence of red‐green color blindness or other uncorrectable visual impairments; (5) alcohol abuse (>50 g/day for men and >20 g/day for women) within 3 months or alcohol intake within 1 week before enrollment; and (6) incomplete demographic information or Stroop results. Additional exclusion criteria for the patients are (7) previous overt HE; (8) upper gastrointestinal bleeding or spontaneous bacterial peritonitis within 3 months before enrollment; (9) previous transjugular intrahepatic portosystemic stent shunt or other shunt surgery; (10) presence or history of malignancies; and (11) heart, lung, or kidney failure or unstable vital signs. The confirmation of cirrhosis was consistent with our previous study.^37^, ^38^

### 4.2 Data collection and definitions of terms

The definition of years of education and the calculation of MELD‐Na are the same as in our previous study.^38^, ^39^ This study assessed QOL using the EQ‐5D questionnaire, which included following five dimensions: mobility, self‐care, usual activities, anxiety or depression, and pain or discomfort. According to the official recommended standards, the questionnaire results were converted into specific scores, ranging from 0.0000 to 1.0000, with a lower score indicating a worse QOL.^40^, ^41^

### 4.3 Stroop test

All subjects completed the Stroop test via EncephalApp on iPad. The test consists of two states named as “off” and “on,” with five runs at both states. Each state is trained twice before starting. In the “off” state, the stimulus is concordance, which means that the subject had to touch the appropriate colors in the “green,” “blue,” and “red” of the symbols presented. The “on” state is more challenging as the select should be discordance with the given word, which means that the subject had to touch the font color of the given words of color name accurately, that is, the word “red” was presented in blue, and the correct response was blue, not red. Every 10 symbols or words were identified as one run in both states. If the subject pressed the wrong color, the run would stop and restart until five runs were completed in each state. In the end, the Stroop test result consists of six parameters: off time, on time, off + on time, off runs, on runs, and on‐off time, which are derived based on total or separate time and number of runs the subjects completed the two states. According to the official recommendation, the off + on time was used to determine MHE.^10^ The model of the expected off + on time value dependent on age, sex, and duration of education was constructed based on the results of healthy people. Patients with off + on time results worse than the expected value calculated by their corresponding characteristics according to the model were diagnosed with MHE.

### 4.4 Psychometric hepatic encephalopathy score

All participants completed the full version of neuropsychological tests to obtain their PHES score.^42^ PHES was recorded and scored in the same way as in our previous study.^38^

### 4.5 Statistical analysis

The presentation of data, comparisons between groups, and correlation testing methods were consistent with our previous study.^38^ Multiple linear regression was performed to construct a model for the expected value of the off + on time based on healthy controls. Statistical significance was set at *p *< 0.05. Statistical analysis was performed using SPSS (version 25.0; IBM) and GraphPad Prism (version 9.0; GraphPad Software).

## AUTHOR CONTRIBUTIONS

S.Y., X.Z., F.M., Y.B., J.L., F.L., C.L., J.L., Q.‐h. Y., W.F., W.Y., J.C., Y.G., C.W., N.W., Q.Z., F. W., J.Y., J.W., X.Y., X.W., Y.L., X.Z., C.W., and W.G. enrolled subjects, and analyzed and interpreted the patient data. X.Q., J.F., F.‐S.W., and J.S.B. participated in research design. X.L., J.F., S.L., Y.G., H.Z., and H.X. contributed to writing of the manuscript. All authors read and approved the final manuscript.

## CONFLICT OF INTEREST STATEMENT

The authors declare no conflicts of interest.

## ETHICS STATEMENT

The study was approved by the Ethics Review group of the Fifth Medical Center of Chinese PLA General Hospital (KY‐2022‐4‐30‐1) and registered for clinical trials (ClinicalTrials.gov identifier: NCT05140837). Written informed consent was obtained from all participants.

## Supporting information

Supporting Information

## Data Availability

The data that support the findings of this study are available from the corresponding author upon reasonable request.
