# Supplementary material for: Detection of minimal hepatic encephalopathy in patients with cirrhosis based on the Stroop‐CN model (NCRCID‐CHESS 2106): a prospective multicenter study
Source: MedComm (2020). 2024 Jul 15;5(8):e627. doi: 10.1002/mco2.627 (PMC11247336; doi:10.1002/mco2.627)
Supplement: Supplementary file 1 — Supporting Information [file MCO2-5-e627-s001.docx]

**Supplementary materials**

**Detection of minimal hepatic encephalopathy in patients with cirrhosis based on the Stroop-CN model (NCRCID-CHESS 2106): A prospective multicenter study**

Short title: Stroop-CN construction and evaluation for MHE

Xiaoyan Li^1,2#^, Shanghao Liu^3#^, Ying Guo^4#^, Hongmei Zu^5#^, Huiling Xiang^6#^, Shaoqi Yang^7^, Xiaoning Zhang^2^, Fanping Meng^2^, Yangzhen Bianba^8^, Jie Li^9^, Fei Liu^10^, Chuang Lei^11^, Jiaojian Lv^12^, Qiao-hua Yang^13^, Wei Fu^14^, Wei Ye^15^, Jiafang Chen^16^, Yanjing Gao^17^, Caiyun Wu^18^, Ningning Wang^19^, Qi Zheng^20^, Fang Wang^21^, Jiali Yu^22^, Jing Wang^23^, Xiaoting Yang^24^, Xiangmei Wang^25^, Yayuan Liu^26^, Xuelan Zhao^27^, Chenxi Wu^28^, Wei Gou^29^, Jasmohan S Bajaj^30*^, Fu-Sheng Wang^1,2*^, Junliang Fu^1,2*^, Xiaolong Qi^31*^

1 Medical School of Chinese PLA, Beijing, 100853, China;

2 Senior Department of Infectious Diseases, The Fifth Medical Center of Chinese PLA General Hospital, National Clinical Research Center for Infectious Diseases, Beijing, 100039, China;

3 The First School of Clinical Medicine of Lanzhou University, Lanzhou 730000, China;

4 Department of Hepatology, the Third people’s Hospital of Taiyuan, Taiyuan, 030000, China;

5 Department of Gastroenterology, the Fourth People’s Hospital of Qinghai Province, Xining, 810000, China;

6 Department of Gastroenterology and Hepatology, Tianjin Third Central Hospital, Tianjin Key Laboratory of Extracorporeal Life Support for Critical Diseases, Institute of Hepatobiliary Disease, Tianjin, 300000, China;

7 Department of Gastroenterology, the General Hospital of Ningxia Medical University, Yinchuan 750004, China;

8 Department of Hepatology, the Third People’s Hospital of Tibet Autonomous Region, Lhasa, 850000, China;

9 Department of Infectious Diseases, Nanjing Drum Tower Hospital, The Affiliated Hospital of Medical School, Nanjing University, Nanjing, 210000, China;

10 Department of Infectious Diseases, Hunan Key Laboratory of Viral Hepatitis, Xiangya Hospital, Central South University, Changsha, 410000, China;

11 Department of Infectious Diseases, The First People’s Hospital of Changde City, Changde, 415000, China;

12 Department of Infectious Disease, Lishui City People’s Hospital, Lishui, 323000, China;

13 Hepatology Department of Infectious Diseases Center, The First People’s Hospital of Huaihua, Huaihua, 418000, China;

14 Department of Hepatology, Shenyang 739 Hospital, Shenyang, 110000, China;

15 Liver cirrhosis treatment center, Nanjing Hospital Affiliated to Nanjing University of Traditional Chinese Medicine, Nanjing, 210000, China;

16 Department of Gastroenterology, Datong city fourth people’s hospital, Datong, 037005, China;

17 Department of Gastroenterology, Qilu Hospital of Shandong University, Jinan, 250012, China;

18 Department of Hepatology, Third People′s Hospital of Linfen City, Linfen, 041000, China;

19 Department of Gastroenterology, The First Hospital of China Medical University, Shenyang, 110000, China;

20 Department of Hepatology, Hepatology Research Institute, the First Affiliated Hospital, Fujian Medical University, Fuzhou, 350005, China;

21 Department of Hepatology, Shenzhen Third People’s Hospital,

National Clinical Research Center for Infectious Disease, The Second

Affiliated Hospital, School of Medicine, Southern University of Science

and Technology, Shenzhen, 518000, China;

22 Department of Gastroenterology, The First Affiliated Hospital of Dalian Medical University, Dalian, 116000, China;

23 Department of Gastroenterology, the Second Affiliated Hospital of Baotou Medical College, Baotou, 014000, China;

24 Department of Gastroenterology, Wuzhong People’s Hospital, Wuzhong, 751100, China;

25 Department of Severe Hepatology, Mengchao Hepatobiliary Hospital of Fujian Medical University, Fuzhou, 350005, China;

26 Department of Gastroenterology, Central hospital of Dandong, Dandong, 118000, China;

27 Department of Gastroenterology Liver Diseases, Chongqing Public Health Medical Center, Chongqing, 404100, China;

28 Liver Disease Diagnosis and Treatment Center, Yiyang Fourth People’s Hospital, Yiyang, 413000, China;

29 Qingdao Sixth People's Hospital, Qingdao, 266000, China;

30 Division of Gastroenterology, Hepatology and Nutrition, Virginia Commonwealth University and Central Virginia Veterans Healthcare System, Richmond, Virginia, USA;

31 Center of Portal Hypertension, Department of Radiology, Zhongda Hospital, Medical School, Southeast University, Nurturing Center of Jiangsu Province for State Laboratory of AI Imaging & Interventional Radiology (Southeast University), Nanjing, China; Basic Medicine Research and Innovation Center of Ministry of Education, Zhongda Hospital, Southeast University, Nanjing, China; State Key Laboratory of Digital Medical Engineering, Nanjing, 210009, China.

# These authors contributed equally to this work.

*** Corresponding authors：**

Xiaolong Qi, MD, Professor of Medicine

Chair, Liver Health Consortium in China (CHESS)

Center of Portal Hypertension, Department of Radiology, Zhongda Hospital, Medical School, Southeast University, Nurturing Center of Jiangsu Province for State Laboratory of AI Imaging & Interventional Radiology (Southeast University), Nanjing, China; Basic Medicine Research and Innovation Center of Ministry of Education, Zhongda Hospital, Southeast University, Nanjing, China; State Key Laboratory of Digital Medical Engineering, Nanjing, China;

E-mail: [qixiaolong@vip.163.com](mailto:qixiaolong@vip.163.com)

Junliang Fu, PhD, MD.

Senior Department of Infectious Diseases, the Fifth Medical Center of Chinese PLA General Hospital, National Clinical Research Center for Infectious Diseases, Beijing, 100039, China;

E-mail: [fjunliang@163.com](mailto:fjunliang@163.com)

Fu-Sheng Wang, PhD, MD.

Senior Department of Infectious Diseases, the Fifth Medical Center of Chinese PLA General Hospital, National Clinical Research Center for Infectious Diseases, Beijing, 100039, China;

E-mail: [fswang302@163.com](mailto:fswang302@163.com)

Jasmohan S Bajaj, MD, MS.

Division of Gastroenterology, Hepatology and Nutrition, Virginia Commonwealth University and Central Virginia Veterans Healthcare System, Richmond, Virginia, USA;

E-mail: [jasmohan.bajaj@vcuhealth.org](mailto:jasmohan.bajaj@vcuhealth.org)

**Supplementary Table 1.** Psychometric hepatic encephalopathy score and Stroop results of test and validation cohort in patients with cirrhosis.

|  | Test (n = 292) | Validation (n = 465) | *p* value |
| --- | --- | --- | --- |
| PHES results |  |  |  |
| NCT-A | 51.03 (38.67, 65.00) | 53.00 (39.89, 75.68) | 0.065 |
| NCT-B | 72.00 (53.36, 102.00) | 72.00 (56.00, 103.83) | 0.522 |
| DST | 32.50 (25.00, 43.00) | 32.00 (22.00, 41.00) | 0.144 |
| LTT-t | 65.31 (50.88,85.73) | 63.13 (48.13, 83.58) | 0.514 |
| LTT-e | 1.50 (0.00, 3.50) | 1.00 (0.00, 4.00) | 0.528 |
| SDT | 64.85 (54.02, 82.00) | 65.38 (51.55, 84.00) | 0.398 |
| Stroop results |  |  |  |
| Off time | 93.017 (80.008, 114.755) | 95.500 (79.085, 120.436) | 0.146 |
| On time | 110.000 (90.056, 130.406) | 108.006 (90.491, 131.411) | 0.836 |
| Off + on time | 204.900 (173.985, 239.297) | 204.992 (174.123, 252.029) | 0.448 |
| Off runs | 6.00 (5.00, 6.00) | 5.00 (5.00, 6.00) | 0.070 |
| On runs | 6.00 (5.00, 7.00) | 6.00 (5.00, 7.00) | 0.378 |
| On – off time | 10.565 (3.272, 22.810) | 10.018 (1.502, 21.086) | 0.209 |
